# Cognitive and Emotional Disturbances Due to COVID-19: An Exploratory Study in the Rehabilitation Setting

**DOI:** 10.3389/fneur.2021.643646

**Published:** 2021-05-17

**Authors:** Caterina Pistarini, Elena Fiabane, Elise Houdayer, Claudio Vassallo, Marina Rita Manera, Federica Alemanno

**Affiliations:** ^1^Department of Neurorehabilitation, Istituti Clinici Scientifici Maugeri, Istituto di Ricovero e Cura a Carattere Scientifico (IRCCS), Pavia, Italy; ^2^Department of Physical and Rehabilitation Medicine, Istituti Clinici Scientifici Maugeri, Genoa, Italy; ^3^Department of Rehabilitation and Functional Recovery, Istituto di Ricovero e Cura a Carattere Scientifico (IRCCS) San Raffaele Scientific Institute, Milan, Italy; ^4^Psychology Unit, Istituti Clinici Scientifici Maugeri, Istituto di Ricovero e Cura a Carattere Scientifico (IRCCS), Pavia, Italy

**Keywords:** rehabilitation, pandemic, depression, stress, cognitive function, COVID-19

## Abstract

The coronavirus disease 19 (COVID-19) can cause neurological, psychiatric, psychological, and psychosocial impairments. Literature regarding cognitive impact of COVID-19 is still limited. The aim of this study was to evaluate cognitive deficits and emotional distress among COVID-19 and post–COVID-19 patients who required functional rehabilitation. Specifically, this study explored and compared cognitive and psychological status of patients in the subacute phase of the disease (COVID-19 group) and patients in the postillness period (post–COVID-19 group). Forty patients admitted to rehabilitation units were enrolled in the study and divided into two groups according to the phase of the disease: (a) COVID-19 group (*n* = 20) and (b) post–COVID-19 group (*n* = 20). All patients underwent a neuropsychological assessment including Mini-Mental State Evaluation (MMSE), Montreal Cognitive Assessment (MoCA), Hamilton Rating Scale for Depression, and Impact of Event Scale–Revised (IES-R). A larger part of the COVID group showed neuropsychological deficits in the total MMSE (35%) compared to the post-COVID group (5%), whereas the majority of both groups (75–70%) reported cognitive impairments in the total MoCA. The post-COVID group reported significantly higher score in MMSE subtests of language (*p* = 0.02) and in MoCA subtests of executive functions (*p* = 0.05), language (*p* = 0.01), and abstraction (*p* = 0.02) compared to the COVID group. Regarding emotional disturbances, ~40% of patients presented with mild to moderate depression (57.9–60%). The post–COVID-19 group reported significantly higher levels of distress at the IES-R compared to the COVID group (*p* = 0.02). These findings highlight the gravity of neuropsychological and psychological symptoms that can be induced by COVID-19 infection and the need for tailored rehabilitation, including cognitive training and psychological support.

## Introduction

In December 2019, a new disease, the coronavirus disease 2019 (COVID-19), emerged in China and rapidly spread over the world, resulting in a global pandemic on March 11, 2020.

Scientific literature suggests that COVID-19 is associated with adverse mental health consequences for general population, hospital staff, and patients, leading to dramatic relapses in the healthcare system worldwide (1, 2).

Several studies confirmed high levels of posttraumatic stress disorder (PTSD) symptoms, distress, anxiety, and depression among COVID-19 patients (3, 4). Because of social isolation, perceived danger, uncertainty, physical discomfort, medication side effects, and fear of virus transmission, patients with COVID-19 may experience loneliness, anger, anxiety, depression, insomnia, PTSD, and stigma (5–7), which could negatively affect individuals' functioning and quality of life (8).

However, most of studies have explored mental health and psychological consequences of patients with COVID-19, and there is a lack of scientific studies investigating the effects of COVID-19 on cognitive functions (9–11).

It is well-known that impairment of cognitive function is common following acute respiratory distress syndrome (ARDS) (12, 13). Cognitive impairment following ARDS has been noted to affect most survivors at hospital discharge, and in ~10% of cases, impairments are persistent at long-term follow-up (12, 14). Neuropsychological impairments may affect memory, attention, and executive functions (14, 15). Furthermore, in severe acute respiratory syndrome (SARS) and Middle East respiratory syndrome (MERS), after recovery from the infection, memory and concentration deficits were found in more than 15% of patients up to 39 months following the infection (5).

It is also noteworthy that the presence of the virus has been found in the cortex and hypothalamus in several SARS patients, as well as edema and neuronal degeneration, lending further support for the theory that coronaviruses may impact the central nervous system (16).

Regarding COVID-19, some studies (5, 10, 17, 18) showed its potential neurological and psychiatric complications, including cerebrovascular events, acute alteration in mental health status (i.e., encephalopathy and encephalitis), and primary psychiatric syndromic diagnoses (i.e., psychosis).

Literature regarding cognitive impact of COVID-19 is still limited. Alemanno et al. (19) analyzed a cohort of 87 COVID-19 patients and showed that ~80% of these patients, in the subacute phase of the disease, showed significant impairments of cognitive functions, including memory, attention, abstraction, and space and time orientation. They also showed that 1 month after hospital discharge, 70% of these patients still showed signs of cognitive dysfunction. Zhou et al. (11) evaluated the impacts of COVID-19 on cognitive functions in recovered patients using neuropsychological tests; their findings suggested a potential cognitive dysfunction in patients with COVID-19 especially in the sustained attention domain. A French study (20) conducted among inpatients with ARDS due to COVID-19 showed that 15 of 45 patients exhibited a dysexecutive syndrome at discharge.

Therefore, there is the need to better investigate the short- and long-term effects of COVID-19 on cognitive functions in order to provide patients with the best care during the acute phase of the disease and with personalized cognitive training after discharge, when needed.

The aim of this study was thus to evaluate cognitive deficits and emotional distress among COVID-19 and post–COVID-19 patients who required functional rehabilitation and were admitted to COVID-19 or post–COVID-19 rehabilitation units. Indeed, it has been shown that ~25% of COVID-19 patients need specialized rehabilitation to address cardiorespiratory, motor, and/or cognitive dysfunctions in the subacute phase of the disease (21). Thus, we aimed to explore and compare cognitive and psychological status of patients in the subacute phase of the disease (COVID-19 group) and patients in the postillness period (post–COVID-19 group).

## Materials and Methods

### Population

This was a cross-sectional and exploratory study. The study population consisted of 40 patients admitted to rehabilitation units in order to optimize their functional status prior to discharge and community reintegration. The sample included two groups of patients, according to the phase of their disease: (a) COVID-19 group (*n* = 20) and (b) post–COVID-19 group (*n* = 20).

The majority of sample developed a severe form of COVID-19, with patients needing respiratory support and presenting cardiorespiratory and neurological complications.

The COVID-19 group included infected patients (positive swab) in the subacute phase of the disease (about 10 days after symptom onset), admitted to the COVID-19 rehabilitation unit of the San Raffaele Hospital (Milan, Italy) from May 7 to May 25. Criteria to admit COVID-19 patients in this unit were as follows: positive swab for SARS-CoV-2, stable Sato_2_ and respiratory rate (RR), no need for respiratory assistance or no more than 2 L/min, absence of fever, and with areas of dependence at the FIM [Functional Independence Measure evaluation (score <100)] (21).

The second group included post–COVID-19 patients admitted to the post–COVID-19 rehabilitation unit of ICS Maugeri Spa SB Institute (Pavia, Italy) from May 8 to August 11, 2020. Criteria to admit post–COVID-19 patients in this unit were two consecutive negative swabs, no ongoing signs or symptoms of COVID-19 infection, stable Sato_2_ and RR, and FIM score <100. The mean time of hospital admission was 25.14 ± 10.39 days after the last negative swab.

Exclusion criteria included (1) history of mental disorders or current treatment for mental illnesses (e.g., antipsychotics, antidepressants, mood stabilizers, antiepileptics, benzodiazepines, and other drugs that may interfere with the assessment); (2) history of neurological diseases that may affect cognitive status; (3) severe physical illnesses that may interfere with the assessment; (4) history of drug abuse or drug dependence; and (5) hearing or visual impairments.

### Procedures

All patients underwent a comprehensive rehabilitation program, tailored based on the patients' clinical features and the stage of the infection and in postinfection. The recovery of COVID-19 and post–COVID-19 patients in rehabilitation units aims at improving the respiratory function, counteracting immobilization, and reducing the rate of long-term complications and disability, to improve cognitive functions and promote psychological health, in order to promote quality of life and community reintegration (22, 23).

During the 1st week of admission, all patients underwent an individual psychological and neuropsychological assessment. Neuropsychological assessment was carried out by an experienced neuropsychologist according to standardized procedures. Patients were individually tested, and the full battery lasted ~30 min. Psychological assessment included the administration of self-report questionnaires. In the COVID-19 rehabilitation units, assessments were performed at patients' bedside to minimize the risks of contagion.

The neuropsychological screening included the Mini-Mental State Examination (MMSE) (24) and the Montreal Cognitive Assessment (MoCA) (25).

The MMSE is the most commonly used test for screening cognitive impairment and consists of a brief 30-point questionnaire. The presence of cognitive impairment was defined by a total score of <23.80 adjusted for age and education in the Italian population (26).

The MoCA is a cognitive screening instrument developed to detect mild cognitive impairment (MCI). It is a simple 10-min paper-and-pencil test and with a maximum score of 30. It assesses multiple cognitive domains including memory, language, executive functions, visuospatial skills, calculation, abstraction, attention, concentration, and orientation. As many other studies (27, 28), we used the original cutoff proposed by the author of the test (25), who recommended that a total MoCA score of <26 indicates the presence of cognitive deficits; furthermore, according to the author, one point should be added to the total score in subjects with a low (<12 years) education level.

Depression was assessed using the Hamilton Depression Rating Scale (29), which is a 17-item semistructured interview assessing depressive symptoms. The items are rated on 3- or 5-point scales, and the total score can range from 0 to 53, with higher scores indicative of higher levels of depression. A total score ranging from 0 to 7 suggests no or minimal symptoms of depression, scores from 8 to 17 indicate mild depression, scores from 18 to 25 suggest moderate depression, and scores of 26 or greater are associated with severe depression.

Psychological distress related to the COVID-19 outbreak was assessed using the Impact of Event Scale–Revised (IES-R) (30), which is a validated 22-item self-report that measures the subjective distress caused by a traumatic event. Patients were asked to rate their level of distress using a 5-point Likert scale ranging from 0 (“not at all”) to 4 (“often”) referring to the previous 7 days. A total score ranging from 0 to 23 indicates the absence of relevant symptoms; from 24 to 32, the presence of mild symptoms; from 33 to 36, the presence of moderate symptoms; and >37, the presence of a severe psychological distress (30, 31).

The present study was approved by the local Scientific Ethics Committee of Maugeri and San Raffaele Hospitals. All participants provided oral and written informed consent to participate in this study.

### Statistical Analysis

Categorical variables were expressed as absolute values (percentage), whereas continuous variables were expressed as mean ± standard deviation. The comparison between the two groups of patients was performed using Mann-Whitney *U*-test for continuous variables, whereas categorical variables were compared using the χ^2^ test. Bonferroni correction for multiple testing was applied. The significance level was *p* < 0.05 (two-tailed). All the analyses were performed using SPSS 22.0 (SPSS Inc., Chicago, IL).

## Results

The total sample for this study included 20 inpatients admitted to a COVID-19 rehabilitation unit and 20 patients admitted to a post–COVID-19 rehabilitation unit in Northern Italy. The mean age of participants was 64.13 ± 11.85 years. Most patients were males (62.5%) with a mean education of 11.15 ± 4.88 years. There were no significant differences for these sociodemographic characteristics between COVID-19 and post–COVID-19 groups. These characteristics are presented in Table 1.

**Table 1:** Socio-demographic characteristics of the study-sample.

	**Total**	**COVID-19 group**	**Post COVID-19 group**		
	**M (SD)**	**M (SD)**	**M (SD)**	**Mann-Whitney U**	***p***
Age	64.13 (11.85)	62.85 (12.35)	65.40 (11.51)	235.00	0.35
Education	11.15 (4.88)	10.65 (5.01)	11.65 (4.82)	217.50	0.64
	**% (n)**	**% (n)**	**% (n)**	***X**^**2**^*	***p***
**Gender**					
Male	62.5 (25)	60 (12)	65 (13)	0.11	0.74
Female	37.5 (15)	40 (8)	35 (7)		

*SD, Standard Deviation. Age and education are reported in years. “n” = absolute value*.

### Cognitive Assessment

Results from the neuropsychological screening tests showed that 35.0% of the COVID group resulted impaired in the total score of MMSE adjusted for age and education (26) compared to 5% of the post-COVID group (χ^2^ = 5.625, *p* = 0.02). We explored any significant differences in cognitive domains of MMSE between the COVID-19 and post–COVID-19 groups (Table 2). We found that the post–COVID-19 group performed significantly better in language (*p* = 0.02).

**Table 2:** Comparison of MMSE subtests between COVID-19 and Post-COVID-19 groups.

**MMSE** ** cognitive domains**	**Range** ** (Min-Max)**	**Total**	**COVID-19** ** group**	**Post COVID-19** ** group**	**Mann-Whitney U**	***p***
		**M (SD)**	**M (SD)**	**M (SD)**		
Temporal orientation	0–5	4.38 (1.19)	4.15 (1.49)	4.60 (0.75)	219.00	0.62
Spatial orientation	0–5	4.43 (0.81)	4.25 (0.96)	4.60 (0.60)	242.00	0.26
Retention	0–3	2.90 (0.38)	2.80 (0.52)	3.00 (0.00)	230.00	0.42
Calculation/attention	0–5	3.98 (1.66)	3.45 (2.11)	4.50 (1.14)	249.50	0.18
Memory recall	0–3	2.15 (1.00)	2.00 (1.12)	2.30 (0.86)	227.00	0.47
Language	0–8	7.34 (1.19)	6.83 (1.50)	7.80 (0.52)	257.00	0.02^*^
Visuospatial	0–1	0.65 (0.48)	0.47 (0.51)	0.80 (0.41)	226.00	0.09
Total score	0–30	25.68 (5.13)	23.80 (6.59)	27.55 (1.79)	246.00	0.22

*M, mean; SD, Standard Deviation.*

* *Significant differences between groups (p < 0.05)*.

**Figure 1:**
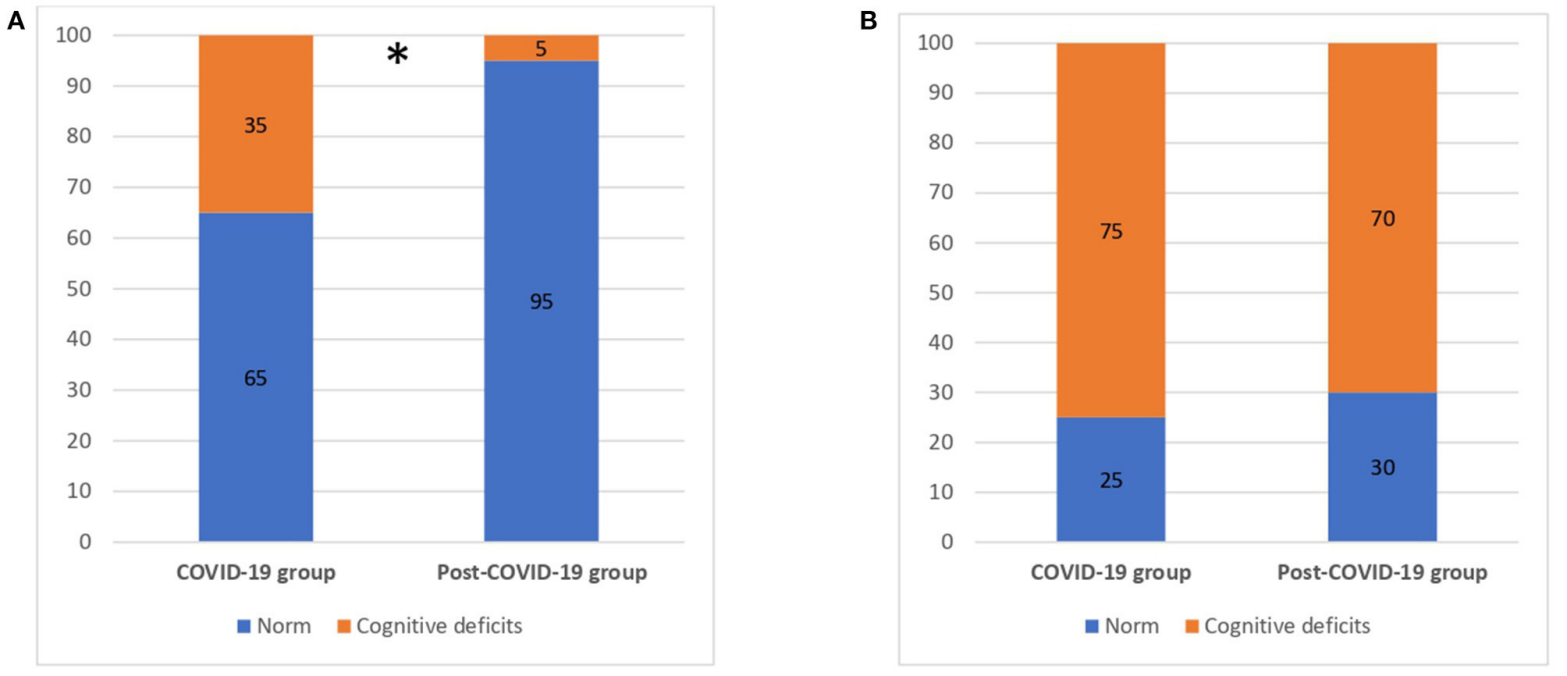
Cognitive deficits for COVID-19 and post-COVID-19 patients. **(A)** MMSE (total score). **(B)** MoCA (total score). MMSE, Mini Mental State Examination; MoCA, Montreal Cognitive Assessment; numbers are reported as %. *p < 0.05.

Regarding the MoCA evaluation, 75.0% of COVID-19 patients and 70.0% of the post-COVID patients showed cognitive deficits according to the MoCA total score adjusted for education (25) with no significant differences between the two groups (*p* > 0.05) (Figure 1).

Table 3 describes the differences between the two groups in cognitive domains measured by MoCA. The post–COVID-19 group reported a significantly better performance in executive functions (*p* = 0.05), language (*p* = 0.01), and abstraction (*p* = 0.016), compared to the COVID-19 group.

**Table 3:** Comparison of MoCA subtests between COVID-19 and Post-COVID-19 groups.

**MoCA** ** cognitive domains**	**Range** ** (Min-Max)**	**Total**	**COVID-19** ** group**	**Post COVID-19** ** group**	**Mann- Whitney U**	***p***
		**M (SD)**	**M (SD)**	**M (SD)**		
Executive functions	0–5	3.05 (1.52)	2.50 (1.76)	3.60 (0.99)	271.50	0.05^*^
Naming	0–3	2.73 (0.68)	2.55 (0.89)	2.90 (0.31)	233.00	0.38
Attention	0–6	4.70 (1.78)	4.30 (2.18)	5.10 (1.21)	230.00	0.43
Language	0–3	1.98 (0.95)	1.55 (0.99)	2.40 (0.68)	297.00	0.01^*^
Abstraction	0–2	1.23 (0.73)	0.95 (0.60)	1.50 (0.76)	282.50	0.02^*^
Delayed recall	0–5	1.83 (1.56)	1.60 (1.72)	2.05 (1.39)	239.50	0.29
Orientation	0–6	5.23 (1.51)	4.70 (1.97)	5.75 (0.44)	255.00	0.07
Total score	0–30	21.97(5.42)	20.12 (7.03)	23.55 (2.89)	200.50	0.35

*M, mean; SD, Standard Deviation*.

* *Significant differences between groups (p < 0.05)*.

### Psychological Assessment

**Figure 2:**
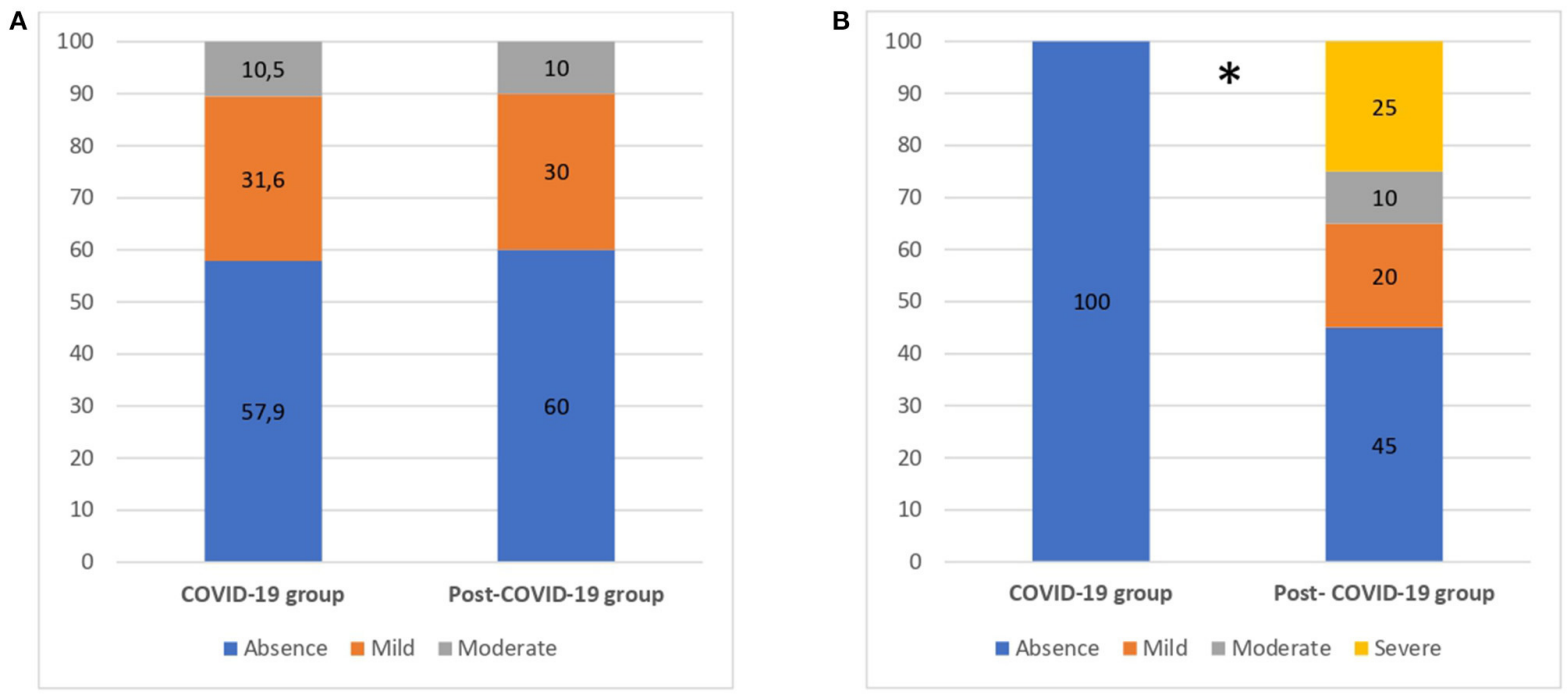
Emotional disturbances for COVID-19 and post-COVID-19 patients. **(A)** Depression. **(B)** Psychological distress. Numbers are reported as %. *p < 0.05.

Figure 2 summarizes the results of depression and psychological distress questionnaires in both groups.

Regarding depression, ~40% of patients, in both groups (57.9%−60%), showed symptoms of mild to moderate depression, with no significant differences between groups (*p* > 0.05). Regarding psychological distress, all of COVID-19 patients (100%) reported an absence of distress. Conversely, 55% of post–COVID-19 patients presented with mild to severe symptoms (χ^2^ = 15.17, *p* = 0.02). Results from psychological questionnaires are summarized in Table 4.

**Table 4:** Comparison of emotional disturbances between COVID-19 and Post-COVID-19 groups.

**Psychological**	**Total**	**COVID-19**	**Post COVID-19**	**Mann-Whitney U**	***p***
**factors**		**group**	**group**		
	**M (SD)**	**M (SD)**	**M (SD)**		
Distress	15.90 (14.43)	9.25 (5.95)	22.55 (17.30)	284.00	0.02^*^
Depression	8.05 (5.60)	7.95 (5.34)	8.15 (5.96)	189.00	0.99

*M, mean; SD, Standard Deviation*.

* *Significant differences between groups (p < 0.05)*.

## Discussion

These results brought further evidence that patients who recovered in COVID and post-COVID functional rehabilitation units presented with cognitive deficits, confirming the results of Alemanno et al. (19). Specifically, 75% of COVID and 70% of post-COVID patients presented with cognitive deficits according to the MoCA evaluation. These data highlight the gravity of neurological and neuropsychological symptoms that can be induced by COVID-19 infection and how these symptoms can outlast the period of infection. Neurological symptoms and cognitive dysfunctions following COVID-19 infection are likely to result from multiple and interacting causes, notably direct damage by the virus to the cortex and adjacent subcortical structures, and indirect effects as a part of broader systemic illness and psychological distress (5, 9). The hippocampus appears to be particularly vulnerable to coronavirus infections, thus increasing the risk of post-infection memory impairment and acceleration of neurodegenerative disorders (9).

In this study, both groups presented with impairment of cognitive functions, such as executive functions, short- and long-term memory, visuospatial abilities, abstraction, and orientation. However, post–COVID-19 patients, ~1 month after infection, showed better performance in the language subdomain, compared to COVID-19 patients, showing incomplete recovery in the 1st days following infection. Although partial recovery could be observed, post–COVID-19 patients still presented with significant memory dysfunctions. Such memory deficits have already been reported in post–COVID-19 patients (11, 19, 30, 32). These data sum up with previous evidence showing that SARS-CoV-2 might affect the nervous system, as also shown by symptoms ranging from loss of smell to increased risk of stroke or delirium (16, 20).

Based on our results, MoCA was more sensitive in detecting cognitive functions impairments, as suggested in previous studies investigating the best tools for MCI diagnosis. Indeed, although MMSE has been widely used to detect cognitive impairments, it would be less efficient than MoCA in detecting MCI (33). MMSE has been further criticized as a poor screening test because of insensitivity to detect visuospatial and executive function impairments (33). MoCA, which includes more testing of cognitive subdomains than MMSE, was thus designed to be more sensitive and may therefore represent a superior screening instrument to detect wide ranges of cognitive impairments.

Regarding the psychological effects of SARS-CoV-2 infection, our data showed that ~40% of patients of both groups presented with mild depression, with no significant differences between groups. Conversely, both groups differed in the distress evaluation. Post–COVID-19 patients presented with higher levels of distress as measured with the IES-R, compared with the subacute COVID-19 patients. Both these results confirm previous evidence reporting a significant amount of depressed patients as well as signs of PTSD in COVID-19 survivors (34). Psychological distress or signs of PTSD were not observed in COVID-19 patients, most probably because patients were still dealing with the infection and were still in the subacute phase of the disease. Indeed, signs of PTSD are usually reported at a certain distance from the stressful events. In this study, COVID-19 patients were still infectious, within a few days from the symptom onset. Thus, it might have been too early to detect, in this subgroup, signs of PTSD. A recent review and meta-analysis of psychiatric and neuropsychiatric presentations associated with SARS, MERS, and COVID-19 (5) showed that during the acute illness, common symptoms among hospitalized patients included confusion, depressed mood, anxiety, and insomnia. PTSD symptoms are commonly observed among patients in the postillness stage with frequent recall of traumatic memories, insomnia, and emotional lability, as confirmed by our results.

Although such cognitive and psychological effects of COVID-19 infection that are reported in this study still need to be better investigated in higher numbers of patients, our results showed that a high majority of recovered COVID-19 and post–COVID-19 patients present with cognitive dysfunctions.

Several factors might have been responsible for such impairments, including systemic inflammation, cerebrovascular changes, and the risk of developing ARDS, which has been highly associated with long-term cognitive impairment, especially in the domains of executive functions and psychomotor tasks (35). Moreover, such results might also have been related to the acute stress induced by patients clinical conditions, which might have, in turn, been related to the degree of invasiveness of oxygen therapy received in the acute phase of the disease (19).

Our data demonstrate the need to perform detailed investigation of cognitive functions in COVID-19 patients or in COVID-19 survivors in order to provide them with cognitive training and psychological support, as soon as possible. More data are also needed regarding the follow-ups of such patients to define the duration of these impairments. Our data already demonstrated that most patients who recovered in the post-COVID rehabilitation unit 1 month after the end of infection still presented with cognitive deficits, confirming the long-lasting duration of these neuropsychological COVID-19 symptoms. The term “long COVID” has been recently introduced to include those patients still suffering from various symptoms weeks or months after the end of infection (36, 37). Long-term symptoms of COVID-19 infections might thus need long-term rehabilitation, including cognitive training. Various modalities can be proposed to patients after home discharge, including telerehabilitation (37). In the last years, telemedicine and telerehabilitation have been progressively expending its fields of application (38, 39). Nowadays, and especially in response to the COVID-19 pandemic, the field of telerehabilitation must rapidly evolve in response to public health concerns and social distancing directives. Cognitive telerehabilitation refers to intensive home-based exercise under the supervision of a clinician via web (i.e., mobile phone with specific health apps or PC-based exercises), including the possibility to adapt the level of difficulty of the exercises to the patient's performance and the possibility to choose different sets of exercises based on the cognitive deficit These characteristics are fundamental to guarantee treatments in a safe manner, create activities tailored to the patient's needs, and improve social functioning and psychological well-being by also avoiding isolation (37).

In this study, we also found high levels of emotional disturbance related to COVID-19, supporting the idea that, during this pandemic, patients would also benefit from telecounseling and telepsychiatry, which refers to any type of psychological service performed over the internet (i.e., counseling, psychotherapy, psychoeducation) (40). Previous studies reported the psychosocial effects of the COVID-19 pandemic on patients, caregivers, and the general population, demonstrating significant increases of issues related to anxiety, depression, and posttraumatic stress syndrome (2–4, 6–8). For example, in China during COVID-19 outbreak, telemedicine mental health services have been used and prioritized for people who are at higher risk of developing severe health complications related to COVID-19, including COVID-19 patients and their families (39).

Some limitations of this study should be mentioned. First, the low number of evaluated patients limits the generalizability of results and findings; however, this represents that an exploratory study and further research are needed to clearly understand cognitive impairment in COVID-19 patients. Second, this study is cross-sectional, with a single cognitive and psychological assessment of patients; it is necessary that future studies explore long-term cognitive end emotional consequences of COVID-19 infection using a longitudinal design. Third, to assess cognitive and emotional disturbances, we used standardized instruments characterized by good psychometric properties and widely used for research and clinical purposes, but the lack of a control group with subjects without COVID-19 infection confirmed by negative serologic tests could limit the interpretation of our results.

To conclude, our data showed extended neuropsychological dysfunctions in patients recovered in functional rehabilitation units for COVID-19 (in the subacute phase of the disease) or post–COVID-19 patients. These data confirmed the potential neurological and neuropsychological sequelae of SARS-CoV-2 infection. Moreover, as many patients with SARS need to recover in intensive care units, such traumatic experience might further increase the likelihood that these individuals may experience neuropsychological dysfunctions during and following their hospitalization. Thus, more attention should be given to the investigation of cognitive functions of COVID-19 patients in order to provide them with adequate cognitive training and subsequent follow-ups, even in the long term, in case of long–COVID-19 syndrome.

## Data Availability Statement

The raw data supporting the conclusions of this article will be made available by the authors, upon reasonable request.

## Ethics Statement

The studies involving human participants were reviewed and approved by the local Scientific Ethics Committee of Istituti Clinici Scientifici Maugeri Spa SB (protocol number: 2470) and San Raffaele Scientific Institute. Written consent was obtained from all participants.

## Author Contributions

CP was responsible for writing the paper and experimental design. EF made data analysis and contributed to writing the paper. CV and MM contributed to experimental design, data collection, and the revisions of the paper. EH contributed to literature review, data collection, and writing the paper. FA was the supervisor of all the process of the research and the preparation of the paper, including comments, and revisions. All authors contributed to the article and approved the submitted version.

## Conflict of Interest

The authors declare that the research was conducted in the absence of any commercial or financial relationships that could be construed as a potential conflict of interest.
